# Current Overview of Osteogenesis Imperfecta

**DOI:** 10.3390/medicina57050464

**Published:** 2021-05-10

**Authors:** Mari Deguchi, Shunichiro Tsuji, Daisuke Katsura, Kyoko Kasahara, Fuminori Kimura, Takashi Murakami

**Affiliations:** Department of Obstetrics & Gynecology, Shiga University of Medical Science, Otsu 520-2192, Shiga, Japan; tsuji002@belle.shiga-med.ac.jp (S.T.); katsuo14@belle.shiga-med.ac.jp (D.K.); kasabee@belle.shiga-med.ac.jp (K.K.); kimurafu@belle.shiga-med.ac.jp (F.K.); tm@belle.shiga-med.ac.jp (T.M.)

**Keywords:** osteogenesis imperfecta, skeletal dysplasia prenatal diagnosis, genetic testing, mesenchymal stem cell transplantation

## Abstract

Osteogenesis imperfecta (OI), or brittle bone disease, is a heterogeneous disorder characterised by bone fragility, multiple fractures, bone deformity, and short stature. OI is a heterogeneous disorder primarily caused by mutations in the genes involved in the production of type 1 collagen. Severe OI is perinatally lethal, while mild OI can sometimes not be recognised until adulthood. Severe or lethal OI can usually be diagnosed using antenatal ultrasound and confirmed by various imaging modalities and genetic testing. The combination of imaging parameters obtained by ultrasound, computed tomography (CT), and magnetic resource imaging (MRI) can not only detect OI accurately but also predict lethality before birth. Moreover, genetic testing, either noninvasive or invasive, can further confirm the diagnosis prenatally. Early and precise diagnoses provide parents with more time to decide on reproductive options. The currently available postnatal treatments for OI are not curative, and individuals with severe OI suffer multiple fractures and bone deformities throughout their lives. In utero mesenchymal stem cell transplantation has been drawing attention as a promising therapy for severe OI, and a clinical trial to assess the safety and efficacy of cell therapy is currently ongoing. In the future, early diagnosis followed by in utero stem cell transplantation should be adopted as a new therapeutic option for severe OI.

## 1. Introduction

Osteogenesis imperfecta (OI) is a rare skeletal dysplasia, with an incidence of 1/15,000–20,000 [1]. The hallmarks of OI are bone fragility, high frequency of fractures, bone deformities, and growth deficiency [2]. As the production of type I collagen in various tissues is impaired, individuals with OI may also suffer from other clinical symptoms such as brittle teeth, blue sclerae, hearing loss, reduced respiratory function, and cardiac valvular regurgitation [2]. The severity of OI varies from mild to extremely severe, with the most severe form being perinatally lethal [3].

The latest technological advances in imaging modalities and molecular diagnostics have enabled the diagnosis of OI as early as during the first to early second trimester. Previously, most cases could only be suspected as OI in the second trimester using ultrasound (US), and definitive diagnosis required invasive genetic testing. Early diagnosis, especially for lethal or severe cases, could provide sufficient time to enhance the parents’ reproductive autonomy in terms of termination of pregnancy, delivery mode, resuscitation after birth, treatment methods, and soliciting genetic counselling for subsequent pregnancies. For this reason, physicians need to become familiar with prenatal diagnostic strategies and provide adequate information for such families. However, owing to the rarity of the disease and rapid advance in technologies, it is challenging to be aware of these diagnostic strategies as well as understand the limitations of them. Therefore, the purpose of this review is to reveal the latest findings regarding foetal diagnosis and perinatal management in OI, with the hope that it will help in perinatal care medicine. This review provides an overview of the current knowledge of OI, primarily focusing on available prenatal diagnostic methods and future perspectives for OI.

## 2. Pathophysiology

The majority of patients with OI have an autosomal dominant mutation in *COL1A1* and *COL1A2*, which encode the α1(I) and α2(I) chains of type I collagen, respectively. More than 1500 dominant mutations in *COL1A1* and *COL1A2* genes have been discovered to date [4,5]. These mutations cause either a structural or quantitative deficiency of type I collagen [5].

Structural and qualitative defects are associated with a more severe phenotype than quantitative defects [2]. Collagen is the main protein in the extracellular matrix of connective tissues [6]. Collagen molecules are composed of three polypeptide chains, which form a triple helix. Glycine is essential for the formation of the triple helix, as only glycine can fit the limited internal helical space [2]. When a genetic mutation causes glycine substitutions, helical flossing is disrupted, which leads to the occurrence of structural and qualitative defects in type I collagen [7]. Substitutions in the α1(I) chains result in lethal outcomes, whereas substitutions in the α2(I) chains are mostly nonlethal [5].

In contrast, haploinsufficiency of *COL1A1* reduces the production of structurally normal collagen, leading to the occurrence of the mildest form of OI [8]. Homozygous null mutations of *COL1A2* result in phenotypes ranging from mild to severe OI, while haploinsufficiency of *COL1A2* generates a normal phenotype.

In addition, there is another rare autosomal dominant mutation in interferon inducible transmembrane protein family 5 (*IFITM5*, also known as *BRIL*) [9]. This mutation leads to the inhibition of differentiation and mineralisation in bone [10].

Most OI cases arise from dominant or sporadic mutations [11]; however, rare autosomal recessive or X-linked mutations have also been identified in the last two decades [2]. Such genes are involved in the extracellular postmodification of collagen (e.g., *CRTAP*, *LEPRE1*, and *PPIB*), collagen folding and intracellular trafficking (e.g., *SERPINH1* and *FKBP10*), ossification or mineralisation (e.g., *SERPINF1*), and osteoblast development (e.g., *WNT1*, *CREB3L1*, and *SP7*) [2,10].

## 3. Classification

The clinical features of OI vary in severity from mild to lethal. In 1979, Sillence et al. proposed four categories of OI based on specific phenotypes [12]. OI type I, which is related to a quantitative deficiency of structurally normal collagen, is the mildest form, which is characterised by blue sclerae but no bone deformities [1]. In contrast, OI types II–IV are caused by structural abnormalities of type I collagen [5]. OI type II is extremely severe and perinatally lethal. OI type III, the most severe form observed in patients who survive the neonatal period, comprises severe progressive deformities and an extremely short stature. OI type IV results in mild to moderate bone deformities, short stature, and normal sclerae [1,7].

With an increase in the discovery of the number of gene mutations responsible for causing OI, the classification of OI subtypes has expanded up to OI type XX to date [13,14,15]. However, even within the same genetic mutations, various phenotypes are observed; therefore, it is difficult to correlate the molecular genetic classification with the Sillence classification [1]. According to the latest International Nomenclature Group of Constitutional Disorders of the Skelton (INCDS), OI is phenotypically divided into five groups, from OI types 1 to 5. Arabic numerals are used for this new classification, so that the original Roman identification still represents molecular and genetic mutations. This classification preserves the Sillence classification, which describes OI types 1 to 4, and adds OI type 5, which is characterised by calcification of the interosseous membrane and is both radiologically and phenotypically distinct from the other four types [14].

## 4. Diagnosis

The clinical diagnosis of OI is based on the clinical features described above. The timing of diagnosis varies according to the severity of OI; it can be during pregnancy, at birth, in childhood, or in adulthood [2].

### 4.1. Prenatal Diagnosis

**Figure 1:**
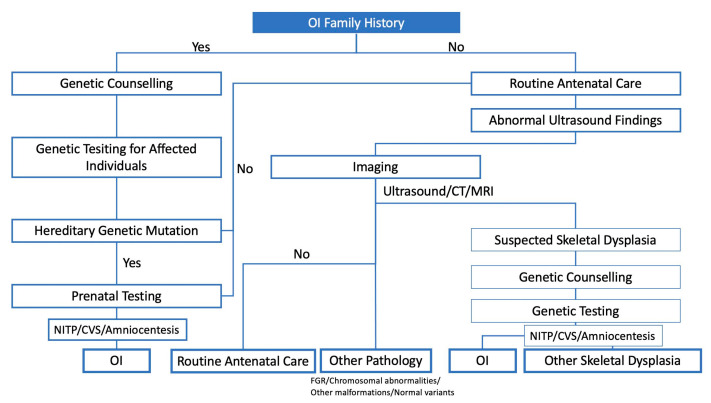
Prenatal diagnostic strategy for OI.

Diagnostic modalities, including US and molecular testing, are essential for the prenatal diagnosis of OI (Figure 1). The diagnostic strategy is roughly divided into two different starting points, that is, in the presence or absence of a family history [16]. When there is an already known familial history of OI, genetic counselling and diagnosis are offered to the affected family, and the evaluation of inheritance patterns (i.e., autosomal dominant or recessive) could provide information to expectant couples. In the absence of a family history, clinicians usually suspect foetal OI during US scanning through the detection of a decrease in the femoral length (FL) in the second trimester [17]. The following section focuses on imaging modalities used for diagnosis.

#### 4.1.1. Ultrasound Evaluation

US examination during pregnancy has become a standard antenatal procedure. Although the scanning schedule varies depending on the country, it is reasonably common to offer at least two scans: one in the first trimester for pregnancy dating and one around 20 weeks to detect congenital anomalies [18]. Because foetal skeletal development starts at eight weeks of pregnancy and secondary ossification centres can be observed at approximately 20 weeks of gestation, second trimester screening is a suitable timing for the prenatal diagnosis of OI and other skeletal dysplasias [19,20].

FL is the most reliable parameter for detecting skeletal dysplasia [21]. Every foetus with FL less than the fifth percentile or two SDs below the mean in the second trimester should be examined by foetal medicine experts, and the following foetal US parameters should be measured for the differential diagnosis of skeletal dysplasia (Table 1) [18,22].

Importantly, physicians should not diagnose skeletal dysplasia based solely on the detection of a low FL because many normal variants or other conditions also show an FL of two SDs below the mean [23,24]. For instance, inaccurate gestational age, intrauterine growth restriction, and chromosomal abnormality can be the differential diagnosis of small FL [17]; therefore, it is essential to obtain precise information and a medical history to rule out other conditions. If physicians feel uncertain of the reason for a short FL, a 3-week-interval follow-up US will demonstrate persistent reduction in femur growth [25]. When the FL is less than four SDs below the mean during follow-up, significant skeletal dysplasia can be suspected [24].

Skeletal dysplasia includes more than 450 disorders [26]. OI is the second most common type of severe skeletal dysplasia. The important differential diagnoses are thanatophoric dysplasia and achondrogenesis, which also include prenatally lethal forms. Thanatophoric dysplasia, the most common lethal skeletal dysplasia, is characterised by extremely short limbs, small chest, macrocrania, frontal bossing, cloverleaf skull, and normal mineralisation without fractures [27]. The other common severe dysplasia is achondrogenesis, the features of which are extremely short limbs, small chest, macrocrania, and decreased mineralisation with occasional fractures [28]. As these various forms of skeletal dysplasia often share the US findings of OI mentioned below, using them as the basis of a differential diagnosis can be difficult [29,30].

Regarding the features of OI, severe and lethal OI is characterised by short limbs, a small chest, no macrocrania, and decreased mineralisation with numerous fractures [29,31,32]. In addition, irregular bending of long bones and limbs is also a known feature of OI, which results from multiple fractures. Moreover, due to severe hypomineralisation, the calvaria are thin and easily compressible [29,31,32].

Distinguishing between lethal OI (type 2) and severe OI (type 3) using US is challenging. Munoz et al. proposed the following criteria for prenatal ultrasonographic diagnosis of lethality: the triad of marked femoral shortening, multiple fractures in a single bone, and ‘demineralisation’ of the calvarium [31]. As shown in Table 2, the defining characteristics of lethal OI are severe demineralisation and absence of posterior acoustic shadowing; these US abnormalities can be detected in early gestation [31]. Previous studies have also shown that the degree of femoral shortening is informative for predicting severity [24,29]. Schramm et al. examined 162 cases of foetal skeletal dysplasia, including 35 cases of OI, and found that the decrease in FL was more remarkable in patients with lethal OI than in those with nonlethal OI, and that the FL Z-score decreased constantly with gestational age [29]. In their study, 89% of patients with severe OI were successfully diagnosed using US [27]. However, even specialists often find it difficult to differentiate between OI types 2 and 3 using US.

#### 4.1.2. Lethality Prediction

Prenatal or neonatal lethality is one of the most significant concerns at the time of US diagnosis. In general, the lethality of skeletal dysplasia is determined mainly by the severity of pulmonary hyperplasia due to thoracic hypoplasia [33]. A chest circumference of less than the fifth percentile for gestational age is one of the indicators of pulmonary hypoplasia [34]; however, small thoracic circumferences do not correlate with lethality [18].

There are two major biometrics associated with lethality: FL-to-abdominal circumference ratio (FL-to-AC ratio) and foetal lung volume [18,35,36,37,38]. Because OI is a rare condition, the criteria for lethality are usually examined in conjunction with other skeletal dysplasias. The following section describes the evidence for these parameters.

Several case series have demonstrated that an FL-to-AC ratio of <0.16 is a reliable predictor of lethal skeletal dysplasia. Rahemtullah et al. surveyed 18 cases of suspected skeletal dysplasia and found that all nine cases of OI where the FL-to-AC ratio was <0.16 were lethal and that those where the ratio was >0.16 were nonlethal [37]. Ramus et al. also demonstrated that the FL-to-AC ratio could predict lethality in cases of undiagnosed skeletal dysplasia. They reviewed the data of 30 foetuses diagnosed with skeletal dysplasia, including four foetuses with OI, and found that neonatal death occurred in 12 of 13 foetuses (92%) with an FL-to-AC ratio of <0.16 [39]. Notably, in their case series, no foetus with a ratio of >0.16 had lethal dysplasia [39]. More recently, Nelson et al. reviewed 45 foetuses diagnosed with skeletal dysplasia; eleven of them, including three patients with OI, showed immediate neonatal death or stillbirth [40]. They reported that those with lethal skeletal dysplasia had a remarkably lower FL-to-AC ratio than did those with nonlethal dysplasia; the corresponding proportions of patients with an FL-to-AC ratio of <0.16 were 91% and 11%, respectively [40]. In addition, they found that foetuses with lethal skeletal dysplasia were more likely to have polyhydramnios than those with nonlethal dysplasia [40]. They concluded that the combination of an FL-to-AC ratio of <0.16 and polyhydramnios can be clinically relevant indicators to distinguish lethality, and that the sensitivity, specificity, and positive and negative predictive values of this cutoff value are 55%, 100%, 100%, and 84%, respectively [40].

Recent studies have shown that foetal lung volumes calculated using three-dimensional US (3D-US) or MRI would be informative for determining lethality in skeletal dysplasia. Hypoplastic lung volume is defined as lung volume below the fifth percentile of that for expected gestational age [36]. Barros et al. reviewed 18 cases of lethal skeletal dysplasia, including four cases of OI, and calculated their lung volumes using 3D-US between 20 and 27 weeks of gestation [36]. They demonstrated that 3D-US detected lethal lung hypoplasia in 15 of 18 foetuses, with a sensitivity of 83.3% and specificity of 100% [36]. They also investigated other 2D-US parameters, such as thoracic circumference, thoracic area-to-AC ratio, and FL-to-AC ratio, all of which had lower sensitivities and specificities for the prediction of lethality than lung volume measured using 3D-US [36].

MRI is also useful for calculating lung volume, especially from the late second trimester to the third trimester, when US is not ideal owing to technical limitations in the late gestational period. The observed-to-expected total foetal lung volume (o/e TLV) measured using MRI is known to be a reliable parameter for the prediction of neonatal survival in congenital diaphragmatic hernia (CDH); the cutoff value for CDH survival is 25% [41,42]. Regarding skeletal dysplasia, Weaver et al. evaluated foetal lung volumes using MRI in 23 foetuses with skeletal dysplasia, including 9 with OI [43]. They confirmed the effectiveness of the measurement of the FL-to-AC ratio as well as o/e TLV. They suggested that an o/e TLV of 47.9% or an FL-to-AC ratio of 0.124 could be a useful cutoff for predicting lethality when evaluating foetal skeletal dysplasia. The sensitivity and specificity of o/e TLV ratios of ≤47.9% were 75% and 82%, respectively. The higher cutoff value of skeletal dysplasia than that of CDH reflects a more complex and multifaceted cause of death in the population [17]. Overall, when the US-based lethality prediction is uncertain, foetal MRI may be considered to evaluate foetal lung hypoplasia in order to predict foetal lethality.

#### 4.1.3. Additional Modalities

- 3D-US and 3D-CT

In the last few decades, 3D imaging modalities have become a helpful and reliable diagnostic modality for the prenatal diagnosis of skeletal dysplasia in addition to the calculation of lung volume [44,45]. Three-dimensional imaging has advantages over 2D imaging in the evaluation of facial dysmorphism, relative proportion of appendicular skeletal elements, and hands and feet [45]. There are two distinct advantages of using 3D imaging: the ability to store images and the ability to rotate the planes [45].

In 2004, Ruano et al. compared the information provided by 2D-US, 3D-US, and 3D helical computed tomography (3D-HCT) in the prenatal diagnosis of skeletal dysplasia [46]. Six patients with skeletal dysplasia, including two with type 2 OI, underwent these three examinations. Two-dimensional ultrasound made an accurate diagnosis in four cases, while both 3D-US and 3D-HCT achieved the correct diagnosis in all six cases. Regarding the identification of abnormalities, both 3D-HCT and 3D-US detected remarkably more abnormalities than did 2D-US (3D-CT: 94.3% [33/35], 3D-US: 77.1% [27/35], 2D-US: 51.4% [18/35]). Another case report also showed that 3D-US and 3D-HCT confirmed fracture deformities of the foetus at 18 weeks of gestation and successfully diagnosed OI type 2 [47]. Three-dimensional ultrasound and 3D-HCT confirmed fracture deformities of the humerus and tibia, suggesting a lethal form of IO [47]. A combination of 3D-US and CT provides more accurate information than 2D-US and enables us to differentiate bone deformities from fractures. In another study, 3D-CT was performed on 19 foetuses with suspected skeletal dysplasia; the diagnosis was confirmed in 17 of them, including three with OI. In their study, 3D-HCT showed 100% sensitivity, specificity, and positive and negative predictive values, while sonography alone had sensitivity, specificity, and positive and negative predictive values of 100%, 85%, 89.5%, and 100%, respectively [48].

For the diagnosis of OI, 3D-HCT is a more reliable modality than US. The major advantages of 3D-US are its cost effectiveness and the absence of radiation exposure. However, the accuracy of the examination is affected by the quantity of amniotic fluid, foetal position, and maternal obesity [17,46]. On the contrary, 3D-HCT can help visualise foetal skeletal structures regardless of foetal position and amniotic fluid volume. By using CT, it is possible to overcome the problems that occur during US scanning. One of the major concerns in performing 3D-HCT is foetal radiation exposure. According to the International Commission of Radiological Protection, foetal radiation exposure of less than 100 mGy would have no practical significance and will not increase lifetime cancer risk [49]. The dose for 3D-HCT was a mean computed tomography dose index (CTDIvol) of 3.5 mGy, which is far below the 100 mGy limit [48]. Overall, 3D-HCT would be a valuable tool for the prenatal diagnosis of skeletal dysplasia, including OI. Considering the risk of radiation exposure, the indications for CT should be considered carefully.

- MRI

MRI has been gaining attention as a second-line imaging modality to confirm US findings for detecting skeletal dysplasia [50,51]. The advantages of MRI include no radiation exposure and better visualisation, even in patients with oligohydramnios [52]. In addition, MRI can help visualise soft tissue lesions, such as those in the cerebrospinal system, which is advantageous for diagnosing diseases such as achondroplasia that cause lesions in the brain tissue [50,51].

In some case series, it has been reported that MRI findings complemented US findings, which led to a more accurate diagnosis of skeletal dysplasia [44,50,51,52]. However, a combination of US and MRI does not show high diagnostic accuracy for OI. For instance, Gilligan et al. reported that the accuracy for prenatal diagnosis of skeletal dysplasia using MRI was 82%, while that of OI was only 27% [44]. Taken together, although MRI may be useful for the detection of lethality by facilitating the calculation of lung volumes as described above and for the diagnosis of other skeletal dysplasias, the contribution of MRI to the prenatal diagnosis of OI is considered to be limited.

### 4.2. Genetic Counselling and Testing

#### 4.2.1. Genetic Counselling

Once imaging modalities indicate a prenatal diagnosis of OI, laboratory investigations are offered to the parents. Genetic counselling is strongly recommended before undergoing any test [53]. When a foetus is diagnosed with lethal OI, termination of pregnancy becomes one of the options. However, 40% of women worldwide are estimated to live in nations where abortion is banned or restricted [54]. If termination of pregnancy is impossible owing to the gestational age or sociocultural system, resuscitation after birth is a significant topic to discuss. These decision-making processes are often ethically and psychologically difficult for pregnant women and their families.

The important role of counselling is to clearly set out options and discuss the risks and benefits with parents. In addition, parents should be provided with balanced information about the abnormalities, and their right to reproductive autonomy should be respected [55]. Even if genetic counselling aims to respect parents’ autonomy, some studies have demonstrated that most parents felt that counselling was directive owing to the attitude of the staff [56]. A study surveyed the effect of shared decision-making consultation sessions on the decision to terminate a pregnancy because of the foetus being diagnosed with beta-thalassaemia, and revealed that women who received this counselling showed a decrease in decisional conflict scores and decisional regret scores [57]. The following section discusses the currently available genetic testing methods for pregnant women.

#### 4.2.2. Noninvasive Prenatal Testing

Noninvasive prenatal testing (NIPT) is a modern technique that utilises circulating cell-free foetal DNA (cffDNA) from maternal peripheral blood for genetic testing [58,59]. In 1997, circulating cffDNA in maternal plasma was discovered using polymerase chain reaction (PCR) to identify Y chromosome-specific DNA sequences [58]. CffDNA can be found at approximately 5 weeks of gestation, and the level of cffDNA increases gradually with an increase in gestational age [60]. The circulating cffDNA is cleared out immediately after delivery, and clearance is complete in all women by 2 weeks postpartum [61]. Therefore, NIPT can be performed as early as at 7 weeks of pregnancy [62].

Since 2011, several companies have begun to provide NIPT for aneuploidies worldwide, which has now been introduced as a common option for prenatal diagnosis in many countries [63]. This concept has been adopted for single-gene disorders as well, as noninvasive prenatal diagnosis (NIPD) has also been developed over the last few decades; however, the progress is slower than that of NIPT for aneuploidy screening due to a much smaller market share and technical difficulties [64,65].

Firstly, NIPD has been developed for autosomal dominant disorders, such as achondroplasia and thanatophoric dysplasia, that are either paternally inherited or occur due to de novo mutations [66]. In these cases, the presence or absence of the mutation in maternal plasma can be diagnostic for the foetal condition [65]. NIPD has also been used for paternal exclusion testing for autosomal recessive conditions, including cystic fibrosis, when the father and mother carry different mutations [65]. NIPD for X-linked conditions or autosomal recessive conditions when the parents carry the same mutation is technically challenging due to the high background of the mutation from maternal cell-free DNA. In these cases, dosage-based techniques such as relative mutation dosage (RMD) and relative haplotype dosage (RHDO) have been utilised [65]. Other than that, bespoke amplicon-based next-generation sequencing assays have also been investigated for several rare monogenic disorders [65]. Multiple other methods have also been utilised for NIPD of genetic disorders, including quantitative real-time PCR, digital PCR, and massively parallel sequencing [64,66,67,68].

Several studies have sought to design an NIPD approach for the detection of common dominant skeletal dysplasias, including OI, and several cases have been successfully diagnosed using these methods [66,67,69,70]. All previous OI cases detected using NIPD harboured *COL1A1* and *COL1A2* variants, making these genes an essential part of the NIPD panel [67,69,70,71]. However, owing to the heterogeneity of OI, diagnosis may be inaccurate if other genes led to OI. Therefore, if there are known mutations in a particular family, bespoke NIPD needs to be suggested as an option for them.

Recently, commercially available NIPT has emerged for single-gene disorders, including OI, such as geneSAFE (Italy) and NATERA (U.S.) [53]. These tests also targeted only the two genes mentioned above. Despite technological and diagnostic limitations, technical developments may allow NIPD to be incorporated into clinical practice in the future, enabling families at risk of OI to have easier access to this test. This may reduce the amount of invasive testing, as discussed below. However, it is arguable whether women with no family history or clinical indication should undergo NIPD; thus, appropriate indications for prenatal testing and counselling are essential. In the current clinical setting, NIPD for the diagnosis of single-gene disorders has been developed for pregnancies at high risk of diseases because of either family history or US findings [72]. 

#### 4.2.3. Invasive Prenatal Testing

Invasive diagnostic approaches such as amniocentesis and chorionic villus sampling (CVS) have seen remarkable developments in the last few decades. They are not only the gold standard for diagnosing aneuploidy but also play an important role in diagnosing other foetal genetic diseases. These invasive methods can harvest enough foetal cells for a standard genetic diagnosis of OI [16,73], and when US findings suggest foetal OI, amniocentesis and CVS are the most commonly used techniques for obtaining a definitive diagnosis. Although various other techniques for genetic diagnosis have been investigated and validated, they are beyond the scope of this review.

The main drawbacks of these invasive tests are physical discomfort for mothers and the possibility of an increased risk of miscarriage [74,75]. However, a recent systematic review revealed that CVS and amniocentesis are not associated with an increased risk of miscarriage over the background risk in women undergoing these procedures [76].

CVS, a biopsy of placental tissue, can be performed between 11 and 14 weeks of gestation [77]. Because the cells sampled by CVS originate from the placenta, there is an associated risk of misdiagnosis due to confined placental mosaicism [78,79,80]. Other than genetic testing, biochemical analysis of type I collagen can also be performed [81]. If collagen screening studies have been conducted on the affected family, foetal OI can be used to culture cells from CVS and analyse collagen products [81].

Amniocentesis requires the insertion of a needle into the uterine cavity and aspiration of amniotic fluid, which has foetal cells originating from the foetal urinary tract and skin [82]. Amniocentesis is typically performed after 15 weeks of pregnancy. A major disadvantage of second-trimester amniocentesis is that the final result is usually available only after 17 weeks of gestation. Such a long waiting period for a diagnosis can be very distressing for couples, particularly because most obstetricians are reluctant to offer surgical termination late in pregnancy [74].

### 4.3. Postnatal Diagnosis

Patients with mild to moderate OI are diagnosed postnatally, based on clinical and radiographic findings already mentioned in the Section 3. [12]. Especially in the mild form of OI, the clinical manifestations can vary even in the same family within the same mutation [1]. Furthermore, it is sometimes difficult to differentiate child abuse or early onset osteoporosis from a mild form of OI [1]. Marlowe et al. investigated the ability of biochemical testing for the identification of children with OI among those in whom abuse was considered. They identified that 4.2% of infants at risk of nonaccidental injury (NAI) were diagnosed with OI [81]. Considering the limited accuracy of a clinical examination to differentiate all children with OI from the situation of suspected NAI, laboratory testing of OI is a helpful tool for precise diagnosis [81].

In addition to skeletal manifestations, various tissues expressing type I collagen are impaired [2]. These includes blue–grey sclera, dentinogenesis imperfecta, young-adult-onset hearing loss, muscle weakness, reduced respiratory function, and cardiac valvular regurgitation [2].These symptoms also aid in the diagnosis of mild OI.

## 5. Management

### 5.1. Mode of Delivery

Previously, although there was no evidence, caesarean delivery was assumed safer and less invasive than vaginal delivery when the foetus was diagnosed with OI [83]. Recent studies have revealed that the delivery mode does not affect the rate of at-birth fractures [84,85].

One study indicated that caesarean delivery neither decreased fracture rates at birth nor increased survival for those with lethal OI [84]. They reviewed 167 cases of maternal and neonatal foetal OI [84]. The overall rate of caesarean section was 54%, and there was an extremely high rate of breech presentation at term (37%). There was no significant difference in new fracture rate and survival between caesarean section and vaginal delivery [84]. The indications for caesarean delivery were nonvertex presentations in 53% of patients and antenatal diagnoses of OI in 15% of patients [84]. Among foetuses with nonlethal OI, the rate of fractures was 40% (24 of 59) among those delivered by caesarean section and 32%(17 of 53) among those delivered vaginally [84].

More recently, a large-cohort systematic analysis also showed that delivery by caesarean section was not associated with a decreased at-birth fracture rate in OI [85]. They surveyed 540 individuals with OI and compared self-reported at-birth fracture rates in individuals with type 1, 3, and 4 OI. They found that 92.6% of type 3 OI, 50.7% of type 4 OI, and 17.2% of type 1 OI cases had at-birth fractures irrespective of the mode of delivery [85]. Additionally, they reported that approximately 40% of OI type 3 cases were breech presentations, which is remarkably higher than the incidence in the general population (5%) [85]. They concluded that a caesarean section should be performed for usual obstetric indications but not solely for the prevention of new fractures [85].

Taken together, there is no evidence that the delivery mode affects the birth fracture rate and prognosis of foetal OI. Caesarean sections should be performed for usual obstetric indications. 

### 5.2. Treatment Options

OI is a systemic and complex disease caused by defects related to various aspects of type I collagen synthesis; hence, a multidisciplinary approach and medical specialist team, which includes an orthopaedic surgeon, endocrinologist, pulmonologist, neurologist, surgeon, radiologist, dentists, and nutritionist, are needed [1,86]. Regarding pharmacological approaches, the use of bisphosphonates is considered the gold standard. Some studies have shown that bisphosphonates contribute to a reduction in fracture rates and bone pain [87]. However, they do not improve the quality of bone and bone pain beyond a year [88,89]. In addition, a recent meta-analysis concluded that the effects of bisphosphonates on the prevention of fractures are inconclusive [90]. 

Furthermore, other pharmacological treatments have been investigated and used for patients with OI. For instance, teriparatide, an anabolic agent that stimulates bone formation, showed a favourable outcome for adult patients with mild OI, increasing the bone mineral density [91,92]. However, teriparatide was not effective for patients with moderate or severe OI [92]. Denosumab, a monoclonal receptor activator that decreases bone resorption, also improved bone mineral density in patients with OI [9,93]. Although the use of denosumab was associated with the risk of hypercalcemia, a clinical trial to evaluate the safety and efficacy of denosumab for OI is currently ongoing [10]. Transforming growth factor beta (TGFβ) inhibition has also been drawing attention as a possible treatment pathway [94]. Based on the results of a preclinical study, a clinical trial is ongoing to evaluate the safety and efficacy of fresolimumab, a TGFβ inhibition antibody, in adult patients with OI [10,94]. Even though various new agents have been investigated, no fundamental cure has been found, especially for severe or paediatric OI.

Recently, many new therapies, including gene therapy and cell therapy, have been developed. The aim of gene therapy is to prevent the expression of mutant alleles [95]. There is a possibility that the use of gene therapy could convert a severe phenotype to a milder form [96]. However, owing to the heterogeneity of OI, it is unlikely that gene therapy itself will become a curative therapy.

On the contrary, stem cell transplantation therapy has also been developed over recent years [97]. The rationale for stem cell therapy is that mutant osteoblasts that produce defective collagen proteins can be replaced with normal cells [98]. Götherström et al. primarily reported two cases of foetal OI treated using in utero foetal MSCs transplantation and postnatal boosting with same-donor MSCs [3]. Both patients were transplanted with human leukocyte antigen (HLA)–unmatched foetal liver MSCs at gestational week 31. One patient with type III OI, who had been observed for 13 years, started to take bisphosphonate for 4 months and was re-transplanted with same-donor foetal liver MSCs at 8 years of age because of an increase in fractures and plateaued growth. After postnatal infusion, the patient did not experience any new fractures for 2 years and experienced improved growth velocity and quality of life. Another patient had been followed for over 6 years and started to take bisphosphonate at 1 month of age. Postnatal transplantation with same-donor foetal liver MSCs was performed at 1.6 years of age, and following the procedure, a resumption of vertical growth was observed. Based on these clinical experiences, the first clinical trial of the transplantation of foetal liver MSCs, the Boost Brittle Bones Before Birth (BOOSTB4) trial is ongoing [99,100]. The BOOSTB4 trial is investigating the safety and tolerability of MSC transplantation as a therapy for severe forms of OI (OI type 3 and 4). Prenatal or postnatal transplantation of MSCs in OI will be a promising and innovative therapy, and further research is expected.

## 6. Conclusions and Future Perspectives

Considering the recent improvement of technology, diagnostic and therapeutic strategies for OI might evolve in the future. This would bring various benefits and cause issues to be solved in prenatal medicine. One of the advantages is that advances in screening and diagnostic technologies enable an earlier, accurate diagnosis of OI. As a result, parents can spend more time deciding their reproductive options [101]. Second, with the development of stem cell therapy, there is a growing possibility that the treatment of OI could be started during pregnancy. Early treatment may enhance the effect of MSC transplantation, as there might be a lesser amount of defective collagen protein that requires replacement [99]. The introduction of MSC transplantation therapy could ameliorate the phenotype especially in patients with severe OI and, thus, improve their quality of life.

However, there are several issues that need to be resolved, including cost issues and ethical issues. Additional modalities, such as CT or genetic testing, are more expensive than the usual US screening methods. In addition, the setting-up of facilities and the training of medical staff, including counsellors, are essential for performing these procedures, because of the lack of cost and resources, the regional disparities might be expanded [101].

So far, while new technologies have advanced the diagnostic and therapeutic procedures, the ethical and legal discussions within each local government and society remain highly undeveloped. Well-trained professional teams are required for the safe and adequate application of prenatal genetic testing and procedures. When patients choose to undergo further prenatal screening, diagnosis, or treatment, we should pay attention not only to the efficacy of technologies but also to their autonomy. The centralisation of clinical facilities might be effective for maintaining the abilities of doctors and the quality of counselling.

## Figures and Tables

**Table 1:** Ultrasound parameters for foetal skeletal dysplasia.

Foetal Ultrasound Parameters
Gestational age
Circumferences (head, abdomen, and chest)
Length of long bones (in all segments of all four extremities)
Shapes of long bones (straight, curved, fractures)
Mineralisation of long bones (echodensity of long bones)
Mineralisation and shape of the cranium
Mineralisation and shape of the vertebrae
Appearance of the metaphyseal ends
Presence of the secondary epiphyses
Shape of the skull (i.e., macrocrania, frontal bossing)
Shape of the thorax (i.e., bell shaped)
Size and shape of the scapula
Foetal face (micrognathia, short upper lips, abnormal shapes of ears)
Hands and feet (foot size, shape, number of digits)
Foetal motion
Abnormal posturing of the extremities
Other congenital anomalies
Evaluation of amniotic fluid volume
Hydrops

**Table 2:** Typical ultrasound findings of severe OI.

Type of OI	Severity	Severity	Ultrasound Findings	Time of Detection
2	Lethal	Perinatally lethal form	Severe demineralisation Brain parenchyma No posterior acoustic shadowing from long bones Long bone shortening Long bone and rib fractures Long bone bowing	14 weeks
3	Severe	Severe progressive deforming form	Long bone shortening Long bone and rib fractures Long bone bowing	18 weeks
